# Antigenic and Genetic Characterization of Swine Influenza Viruses Identified in the European Region of Russia, 2014–2020

**DOI:** 10.3389/fmicb.2021.662028

**Published:** 2021-04-15

**Authors:** Daria M. Danilenko, Andrey B. Komissarov, Artem V. Fadeev, Mikhail I. Bakaev, Anna A. Ivanova, Polina A. Petrova, Anastasia D. Vassilieva, Kseniya S. Komissarova, Alyona I. Zheltukhina, Nadezhda I. Konovalova, Andrey V. Vasin

**Affiliations:** ^1^WHO-Recognized National Influenza Centre (NIC), Smorodintsev Research Institute of Influenza, Ministry of Health, Saint Petersburg, Russia; ^2^Peter the Great St. Petersburg Polytechnic University, Saint Petersburg, Russia

**Keywords:** swine influenza, surveillance, HI assay, sequencing, reverse zoonosis

## Abstract

Pigs have long been recognized as “mixing vessels” in which new viruses are formed by reassortment involving various influenza virus lineages (avian, animal, human). However, surveillance of swine influenza viruses only gained real significance after the 2009 pandemic. A fundamentally important point is the fact that there is still no regular surveillance of swine flu in Russia, and the role of swine viruses is underestimated since, as a rule, they do not cause serious disease in animals. Since the pig population in Russia is large, it is obvious that the lack of monitoring and insufficient study of swine influenza evolution constitutes a gap in animal influenza surveillance, not only for Russia, but globally. A 6 year joint effort enabled identification of SIV subtypes that circulate in the pig population of Russia’s European geographic region. The swine influenza viruses isolated were antigenically and genetically diverse. Some were similar to human influenza viruses of A(H1N1)pdm09 and A(H3N2) subtype, while others were reassortant A(H1pdm09N2) and A(H1avN2) and were antigenically distinct from human H1N1 and H1N1pdm09 strains. Analysis of swine serum samples collected throughout the seasons showed that the number of sera positive for influenza viruses has increased in recent years. This indicates that swine populations are highly susceptible to infection with human influenza viruses. It also stresses the need for regular SIV surveillance, monitoring of viral evolution, and strengthening of pandemic preparedness.

## Introduction

The ability to monitor, identify and predict infectious disease outbreaks caused by zoonotic pathogens relies heavily on understanding their ecology and evolution. Swine influenza viruses are known to be the “mixing vessels” wherein new influenza viruses emerge through a process of reassortment between avian, human, and animal influenza virus genes (Castrucci et al., 1993; Ma et al., 2008; Nelson and Worobey, 2018). Although the WHO Global Influenza Surveillance and Response System (GISRS) has conducted precise monitoring of human influenza virus evolution for the last 70 years, less effort has been concentrated on other influenza hosts (World Health Organization, 2019). Currently, there is active global monitoring of avian influenza under the aegis of the WHO and public veterinary organizations (FAO/OFFLU), which also carry out periodic monitoring of influenza viruses in animals, including swine. Global experts have called for increased swine influenza virus surveillance, including detailed systematic analysis of pathogen properties, in order to identify viruses posing a potential threat to humans in a timely manner (Ma et al., 2009; Nelson et al., 2012; Nelson and Vincent, 2015).

Interestingly, our knowledge and understanding of influenza virus biology goes back to swine isolates. The first swine influenza A virus was isolated in the United States in the 1930s. Antigenically, this virus was similar to the human influenza A (H1N1) virus circulating at that time and was termed “classic” swine flu (Schultz-Cherry et al., 2013). Export of animals from the United States to Europe led to the virus first entering the European continent in 1976 and rapidly spreading in the pig population. In 1979, a virus of wild duck origin and antigenically distinct from the “classic” swine A(H1N1) strain was identified in Europe (Zell et al., 2013). This avian strain rapidly and completely supplanted “classic” swine flu viruses circulating in the European continent and also spread in Asia. These two lineages, classic H1N1 and avian H1N1, were the main ones circulating in swine until the 1990s. In the 1980s, reassortant H1N2 viruses were detected in England, and then in Europe, giving rise to new swine influenza lineages (Brown et al., 1998).

Since then, swine influenza viruses have undergone multiple reassortment events that have shaped future diversity. In 1998, swine influenza A (H1N1) triple reassortant viruses became widespread in the United States. The wide distribution of triple reassortants across the US has been detailed in numerous swine influenza surveillance studies (Webby et al., 2000; Rajão et al., 2017). In early 2009, or slightly before, these viruses again underwent reassortment, this time with influenza A viruses of the 1979 Eurasian swine influenza line. As a result, a strain known as the pandemic influenza virus, or A (H1N1) pdm09, has spread rapidly around the world and caused illness in millions of people. At the end of 2009, researchers detected viruses in Hong Kong that were genetically similar to pandemic influenza A (H1N1) pdm09 viruses in seven of the eight segments (Smith et al., 2009). However, the time of most recent common ancestor (tMRCA) showed that these viruses differ in timing from the causative agent of the pandemic by at least 10 years.

As with the pandemic 1918 H1N1 virus, the pandemic influenza A(H1N1)pdm09 virus has been repeatedly transmitted from humans to swine, resulting in multiple events of reverse zoonosis (Nelson and Vincent, 2015). This host jump resulted in generation of multiple reassortant lineages in swine influenza viruses circulating worldwide, with at least 23 genotypes identified in Europe by 2013 (Watson et al., 2015). Many of these new reassortant strains cause sporadic illness in humans. Given the proven pandemic potential of swine influenza viruses, global implementation of regular surveillance for these pathogens is crucially needed, especially in regions featuring dense pig populations (Chauhan and Gordon, 2020).

Russia holds fifth place globally in pork production, mainly grown for the local market. The volume of imported pork has been growing in recent years, but export volume is low (<1%). Currently, there is no systematic surveillance of swine influenza performed in Russia. The role of these viruses is underestimated due to the fact that they do not generally cause serious illness in swine. Only scarce research is available, and only 15 genetic sequences of swine influenza strains are available at GISAID at the moment (apart from this study). The swine herd population in Russia is huge, and it is obvious that the lack of swine influenza research and surveillance of these mammals is a gap. Gaining insight into the swine influenza virus diversity circulating in the country would be valuable not only at the local level, but globally as well. This article summarizes the results of a 6 year collaborative study aimed at identifying influenza virus characteristics in swine herds in the European region of Russia. We identified a dominance of the H1 subtype over H3 swine influenza A viruses. All identified swine influenza A strains were sensitive to adamantanes and neuraminidase inhibitors and did not bear genetic markers known to be associated with increased pathogenicity. Serological studies identified wide circulation of influenza strains in the pig population in the European region of Russia. Thus, increased swine influenza surveillance is important to understand trends in swine influenza spread, including possible emergence of other genetic lineages of the pathogen, in order to enable timely detection of potentially pandemic influenza strains.

## Materials and Methods

### Data Collection

The study took place between February 2014 and November 2020. Biological samples were obtained in two ways: from pig production sites with animals showing clinical signs of illness; and from production sites not registering any clinical signs of illness (who gave consent to perform influenza virus screening). All pig production sites were located in the European region of Russia. Geographically, hog production facilities are mostly densely concentrated in this area. Two types of animal samples from production sites were collected: nasal swabs and blood sera. Nasal swabs were collected in universal transport medium UTM-330C (Copan, Italy) with two flocked swabs per animal. Samples were stored at +4°C during transport (if shipping could occur within 48 h of collection), or frozen. Blood samples for serum were collected in vacutainer tubes with clot activator. Serum samples were then transferred to sterile microtubes and were frozen prior to transportation and storage (before analysis).

Samples from sites where animals did not display clinical signs of infection were primarily serum samples from animals of different age groups. Nasal swab and blood sample collection was the same as described above.

### Processing of Nasal Swabs

All procedures with samples were carried out according to the National Biosafety standards in BSL-2 laboratories. Upon arrival for analysis, Copan tubes with flocked swabs were centrifuged for 10 min at 3,000 rpm/min. The resulting supernatants were filtered through a 0.45 um syringe filter, and then through a 0.22 um syringe filter, to remove possible bacterial or fungal contamination for further PCR testing and virus isolation. Viral RNA was extracted using a commercial kit (RNeasy mini, QIAGEN) according to the manufacturer’s protocol. Detection of viral RNA was performed by real time RT-PCR using Ambion AgPath-ID One-Step RT-PCR Reagent (Applied Biosystems). Primers and probes (targeting the M gene of influenza A) were obtained from the CDC (Atlanta, United States). All PCR-positive samples were further processed for virus isolation.

### Virus Isolation

76 PCR-positive swabs were further processed for virus isolation. Madin-Darby canine kidney cells (London line, obtained from WHO CC World Influenza Centre, London) were used for isolation of influenza A viruses (IAV) from rRT-PCR positive samples. Briefly, cells were seeded on Nunc cell culture tubes (Nunc, Thermo Fisher Scientific) 1 day before inoculation to form a 90–95% confluent monolayer. Cells were washed twice with MEM media containing 2 μg of TPCK-trypsin and penicillin-streptomycin (10,000 units and 10 mg/ml, respectively), and 200 μl of virus-containing media was inoculated into each tube. Tubes were kept at 36°C for 40 min to allow virus absorption. Then 1.8 ml of virus growth media was added [MEM containing 2 μg of TPCK-trypsin and penicillin-streptomycin, bovine albumin fraction V (2.6 ml per 100 ml, Sigma Aldrich), HEPES buffer (1.6 ml, Sigma-Aldrich)]. Tubes were kept for 3–6 days at 36°C and monitored daily for progression of CPE. Once CPE was detected, the tubes were frozen at −80°C, thawed, and the hemagglutination assay with human (group O) red blood cell suspension (0.75%) was performed to determine viral titers. Isolated viruses were further sequenced.

### Antigenic Analysis of Isolated Influenza Strains by Hemagglutination Inhibition Assay

Post-infection rat immune sera were raised to a panel of A(H1N1), A(H1N2), and A(H3N2) viruses including A(H1N1)pdm09 viruses. Antisera were harvested from rats 14 days post-infection following an intramuscular triple immunization with virus-containing cell supernatant (HA titer of virus > 1:64). Diagnostic hyperimmune antisera from the WHO kit, distributed through IRR, were also used.

HI assays using rat polyclonal antisera raised against swine and human influenza A strains were performed to compare the antigenic properties of swine and human influenza H1 and H3 viruses. All antisera were treated with receptor-destroying enzyme (Denka Seiken, Japan) and then heat inactivated at 56°C for 30 min. All antisera were checked with 0.75% human red blood cells (RBC) for the presence of non-specific inhibitors of hemagglutination. HI assays were performed by testing reference antisera, raised against selected influenza viruses (H1 swine; H1 and H3 human), according to standard techniques. Serial twofold dilutions of antisera, starting at 1:10, were tested for their ability to inhibit the agglutination of 0.75% human RBC with four hemagglutinating units of swine and human influenza A viruses.

Hemagglutination inhibition assay for detection of antibodies against influenza A viruses in swine serum samples.

1534 swine serum samples were collected and stored as described above. After thawing, each serum was treated with receptor-destroying enzyme (RDE) for 18 h at 37°C, heat inactivated at 56°C for 30 min, followed by adsorption with chicken red blood cells. A panel of A(H1N1), A(H1N2), and A(H3N2) swine and human influenza A viruses, grown in chicken embryos (vaccine strains) or MDCK culture (reference strains), were selected to assess the presence of influenza A antibodies in pig serum samples. All antigens were standardized to 8 HAU/50 μl prior to 30 min of incubation (room temperature) with twofold serial dilutions of antiserum (starting dilution 1:10). Chicken red blood cells (0.5% in physiological saline) were used to evaluate hemagglutination inhibition.

### Antigenic Cartography

Antigenic cartography was performed using online software^1^ as described (Smith et al., 2004). The results are presented as antigenic maps reflecting the antigenic distances between reference and test antigens. The H1N1pdm09 antigenic map was built using the data on 131 influenza viruses (6 swine influenza A viruses and 125 human influenza strains) and 11 reference antisera; the H3N2 antigenic map was generated using analysis of 144 influenza strains (4 swine influenza strains and 140 human influenza strains) and 13 reference antisera. The spheres in the maps (representing viruses) are color coded as reference antigens, human influenza strains, or swine influenza strains.

### Sequencing

Viral RNA was extracted using QiaAmp Viral RNA mini kit (Qiagen, Germany)RT-PCR for whole genome amplification was performed according to Zhou et al. (2009). RT-PCR products were purified using AmPureXP magnetic beads (BeckmanCoulter, United States). Qubit spectrofluorimeter was used to measure the concentration of purified DNA.

### Sanger Sequencing

For Sanger sequencing of Influenza A (H1N1)pdm09 viruses WHO primer set was used. Sequencing was performed using BigDye 3.1 Cycle Sequencing kit (Thermo Fisher Scientific, United States) on 3130 Genetic Analyzer (Applied Biosystems, United States). Vector NTI software was used for sequence assembly.

### NGS Sequencing

Libraries for Illumina sequencing were prepared using Nextera XT library preparation kit (Illumina, United States) and then sequenced on MiSeq instrument (Illumina, United States) with MiSeq Sequence kit v3. FastQC software was used for sequence data quality assessment. Trimmomatic was used for quality data trimming. Reads were mapped onto reference sequence using BWA. Consensus sequence was obtained using Samtools mpileup.

Libraries for Oxford Nanopore sequencing were prepared using DNA Ligation Sequence kit SQK-LSK109 (Oxford Nanopore, United Kingdom). Sequencing was performed on MinIon instrument (Oxford Nanopore, United Kingdom) with R9.4.1 flowcell. Guppy software was used for basecalling and data quality trimming. Reads were mapped onto reference sequence using Minimap2. Consensus sequence was obtained using Samtools mpileup.Phylogenetic analysis.

Phylogenetic trees were built for the HA and NA genome segments. Swine, seasonal, and pandemic human influenza virus sequences were downloaded from the GISAID for the analysis. For human influenza viruses, the following criteria were used: (1) full genome segments; (2) virus type A; (3) A(H1N1), A(H1N1)pdm09, or A(H1N2) subtype; and (4) reference of vaccine strain. For swine influenza viruses, all available full-genome sequences for HA and NA were downloaded and analyzed. Sequences for each included strain were aligned using the Muscle application within MEGA7.0 software. Maximum-likelihood phylogenetic trees were built using MEGA7.0 with 1000 bootstraps using the general time-reversible model (GTR) with gamma rate categories. Secondary analysis was performed using MAFFT and IQ-tree to confirm the locations and fidelity of the represented ML phylogenetic tree topologies.

## Results

### Overall Description of Positive Cases

Table 1 summarizes the results for samples obtained from production sites over the investigation period. Overall, 1534 serum samples were tested for serological evidence of influenza A antibodies; approximately half of them came from sites with no clinical signs of illness. All swabs in VTM obtained from production sites with no evident clinical signs of infection in pigs were PCR-negative or had high Ct values (>36); they were not tested further. More samples came from sites where animals had clinical sings of illness. However, only a small fraction of swabs were PCR positive for IAV (17.7%, 76 samples); those were detected in 2014, 2017, and 2019. Analysis of samples obtained in other years did not reveal positive samples.

**TABLE 1:** Numbers of samples received from production sites.

**Year**	**# of samples from sites with no signs of disease**		**# of samples from sites with evident signs of disease**	
	**sera**	**swabs**	**sera**	**swabs**
2014	−	100	−	100
2016	200	69	100	111
2017	300	95	293	70
2018	150	−	103	25
2019	−	−	225	100
2020	60	−	103	21
Total	710	264	824	427

### Virus Isolation and Characterization

All 76 PCR-positive for influenza A samples were processed for influenza virus isolation. The overall isolation rate was 21%: 16 influenza viruses were isolated. Isolated influenza strains were typed by HI assay with the CDC diagnostic kit. Of eight influenza strains isolated from samples collected in 2014, three belonged to the H1pdm09 subtype; five strains were of the A(H3) subtype (Table 2).

**TABLE 2:** Number of influenza strains isolated from IAV-positive samples (by rRT-PCR).

**Year**	**Total # of swabs**	**IAV positive swabs**	**Number of isolated strains (subtype)**
2014	100	62, of these 15 A(H1N1)pdm09, 18 A(H3N2)	3 H1N1pdm09 5 H3N2
2016	111	−	−
2017	70	6, all H1	3 H1N2
2018	25	−	−
2019	100	8, all H1	2H1pdm09 3H1avN2
2020	21	−	−
Total	427	76	16

**av—avian lineage of swine influenza viruses.**

**FIGURE 1:**
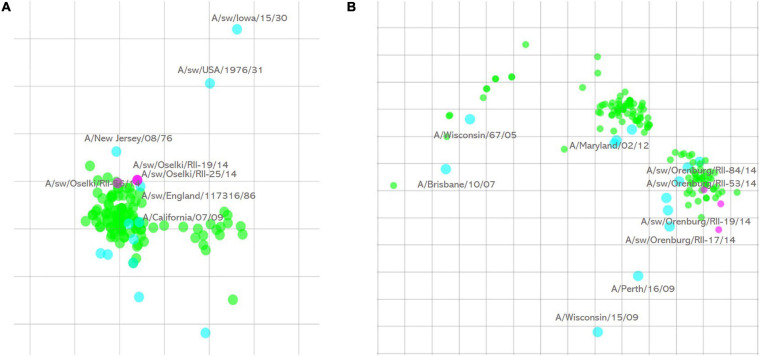
Antigenic cartography of swine influenza A(H1N1)pdm09 **(A)** and influenza A(H3N2) **(B)** strains isolated in 2014. One square of the antigenic maps is equal to a twofold difference in HI titer. Green spheres—test antigens, cyan spheres—reference antigens, magenta—swine influenza strains isolated in this study.

Subsequent HI assay of swine influenza viruses isolated in 2014 with the panel of antisera raised against reference human and swine influenza A strains showed that all of them were human-like viruses. All three influenza A(H1N1)pdm09 were well recognized by antisera raised against reference and vaccine human influenza A(H1N1)pdm09 strains. Antigenic cartography of these viruses (Figure 1A) shows that these swine strains formed a single antigenic cluster with human influenza viruses isolated during the 2010–2011 influenza season. Human influenza strains isolated in 2013–2014 and included in the analysis formed a separate cluster on the map indicating slower antigenic drift of H1N1pdm09 viruses in swine in that time period.

Influenza A(H3N2) strains were only isolated in 2014. Five isolated strains of this subtype were antigenically similar, and four of them are presented on the antigenic map (Figure 1B). Antigenic analysis of these swine influenza strains, along with human influenza A(H3N2) viruses isolated in 2008–2014, showed that swine influenza strains were outliers while human influenza strains formed visible tight clusters on the antigenic map. Sequencing results (see below) show that they have an amino acid substitution in the HA1 antigenic site that causes their separate grouping on the map.

Influenza strains could be isolated from swabs only in 2017. Three influenza strains were isolated from PCR-positive swabs: A/swine/Leningrad region/RII-02/2017; A/swine/Leningrad region/RII-03/2017; and A/swine/Leningrad region/RII-06/2017. All of them were subtyped by HI assay as influenza A(H1)pdm09 strains. However, antigenic analysis of these strains showed that they were not antigenically related to A/California/07/09 (Table 3).

**TABLE 3:** Antigenic characterization of swine influenza A viruses by HI assay.

**Influenza virus strain**	**CDC diagnostic antisera**			**Polyclonal rat antisera**							
	**Í1pdm09 FR 1682**	**H1 seasonal Lot 98-0045 A/Tw/1/86**	**H1seasonal Lot 03-0322 A/NC/20/99**	**Hu A/Tw/1/86**	**Hu A/NC/20/99**	**Hu A/Cal/7/09**	**Classic SW H1N1**	**SW H1N2**	**SW H1N1 Avian**	**SW H1 A/sw/Oselki/56/14**	**SW H1 A/sw/Siberia/1/16**
A/Taiwan/1/86	40	1,280	80	**320**	<	<	<	160	<	<	40
A/New Caledonia/20/99	<	640	1,280	10	640	<	<	<	<	<	<
A/California/7/09	1280	160	160	160	<	**320**	<	20	40	80	<
A/sw/England/117316/1986 Classical sw H1N1	640	40	<	40	<	640	**640**	<	80	80	20
A/sw/England/438207/1994 H1N2	<	1,280	1,280	40	<	<	<	**1,280**	<	<	320
A/sw/England/195852/1992 H1avN1	<	40	320	<	<	<	<	10	**160**	<	10
A/sw/Oselki/56/14	20	n/d	n/d	40	40	320	<	<	<	**320**	<
A/sw/Siberia/01/16	320	1,280	640	320	160	40	40	640	40	40	**640**
A/sw/Leningrad region/02/2017	320	320	320	160	<	40	20	20	10	40	20
A/sw/Leningrad region/03/2017	640	320	320	80	<	40	20	40	10	80	20
A/sw/Leningrad region/06/2017	320	320	320	80	<	40	20	20	10	80	20
A/sw/Pskov region/RII-06-100/2019	160	20	40	10	<	40	<	20	40	20	40
A/sw/Pskov region/RII-08-100/2019	80	10	40	20	<	40	<	20	40	20	40
A/sw/Pskov region/RII-41-2/2019	160	<	20	20	<	40	10	40	40	20	40
A/sw/Pskov region/RII-PR1/2019	160	<	40	80	<	40	40	10	10	40	10

*The homologous titres of the reference antisera are indicated in bold.*

In 2019, five other influenza A(H1) strains were isolated, and these were of two different antigenic lineages. Two of these viruses (A/swine/Pskov region/RII-PR1/2019, A/swine/Pskov region/RII-PR8/2019) were recognized by A(H1)pdm09 diagnostic antisera. They were also better recognized by antisera raised against the A/swine/Oselki/RII-56/2014 and A/swine/England/117316/1986 (classical swine H1N1) viruses. Three other strains (A/swine/Pskov region/RII-6-100/2019, A/swine/Pskov region/RII-8-100/2019, A/swine/Pskov region/RII-41-2/2019) were also recognized by the A(H1)pdm09 diagnostic antisera, but reacted poorly with the antisera raised against A(H1N1)pdm09 and A(H1N1) classical swine strains. They were better recognized by antisera raised against the A/sw/England/195852/1992 (H1 avian-like) and A/swine/Siberia/01/2016 strains, indicating antigenic difference of these two groups of strains. It is worth noting that all of these strains were isolated from samples collected from one production site. This indicates that multiple swine lineages can be co-circulating and infecting pigs at the same time.

### Phylogenetic Analysis

**FIGURE 2:**
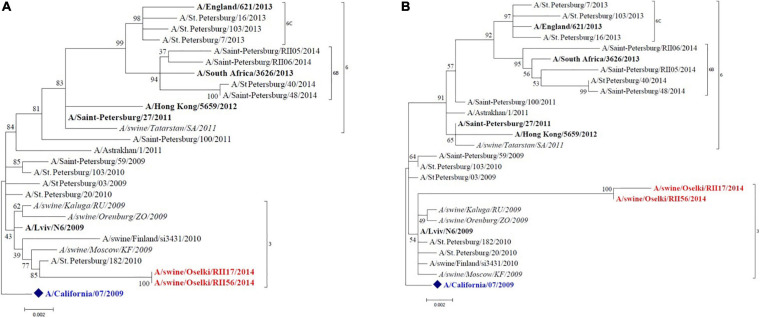
Maximum likelihood tree for swine influenza A HA **(A)** and NA **(B)** of H1N1pdm09 isolated in 2014. The vaccine strain at that time is shown in blue. Reference strains are in black bold. Swine strains from this study are shown in red.

Full-genome sequencing was performed for 15 influenza strains isolated in 2014, 2017, and 2019 to allow determination of genetic lineages of influenza A viruses isolated from swine. Figure 2 shows the HA and NA phylogenetic trees of the A(H1N1)pdm09 swine viruses isolated in 2014. These viruses clustered with human influenza A(H1N1)pdm09 viruses of genetic group 3 that widely circulated in humans in that period of time. The NA of these isolates also groups with the same strains. The tree shows other A(H1N1)pdm09 swine strains identified in Russia that are closely grouped with swine strains isolated in this study; this possibly indicates multiple reverse zoonosis events since the emergence of A(H1N1)pdm09 in the human population.

**FIGURE 3:**
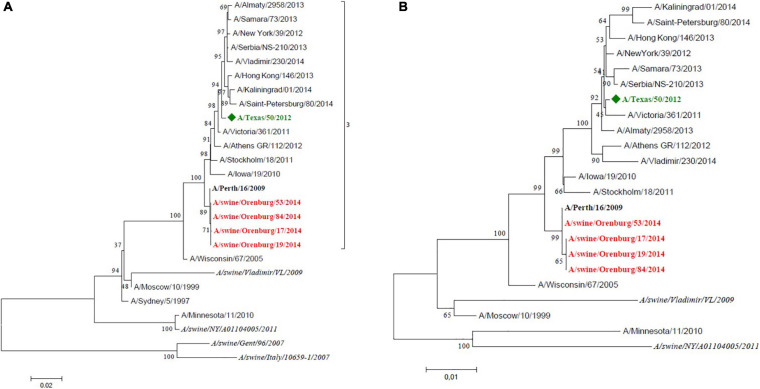
Maximum likelihood tree for swine influenza A HA **(A)** and NA **(B)** of H3N2 isolated in 2014. The vaccine strain at that time is shown in green. Reference strains are in black bold. Swine strains from this study are shown in red.

Swine influenza A(H3N2) samples were genetically closely related to human influenza vaccine strain A/Perth/16/09 (Figure 3); they are tightly clustered with this strain on the tree. However, the antigenic analysis data showed some discrepancy with the phylogenetic tree. This was due to a key amino acid change in HA (position 156, H→Q) which lies in antigenic site B and is associated with cluster/outlier transitions in swine H3 wild-type viruses (Abente et al., 2016).

The three 2017 isolates from the Leningrad region (RII-02, RII-03, RII-06) were attributed to H1pdm09N2, with all segments from pandemic 2009 human influenza virus except the neuraminidase. These three strains clustered together on the HA phylogenetic tree with the previously isolated A/swine/Oselki/RII-56/2014 strain and other swine influenza strains of H1pdm09 subtype. Detailed HA sequence analysis of these strains showed that all of them feature D168N amino acid substitutions located at the Ca antigenic site, along with S183P. This explains their poor recognition by antisera raised against the A/California/07/2009 strain and other antigenically related strains (Tapia et al., 2020).

**FIGURE 4:**
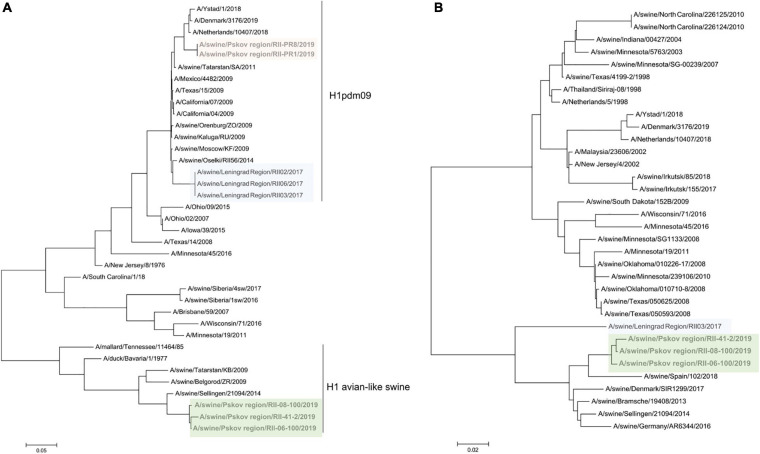
Maximum likelihood tree for swine influenza A HA **(A)** and NA **(B)** of reassortant H1N2 viruses isolated in 2017 and 2019. H1pdm09-like HA are shaded in pink and blue, avian-like H1 are shaded in green.

Strains from 2019 formed two distinct lineages. Two isolates from the Pskov region (RII-PR1, RII-PR8) were A(H1N1)pdm09 viruses with all gene segments from pandemic 2009 influenza. These two isolates grouped closely with a human reassortant A(H1pdm09N2) strain from the Netherlands belonging to the 6B.1 genetic subclade (Figure 4). Human influenza viruses of genetic subclade 6B.1 are known to be well recognized by antisera raised against the A/California/07/209 strain, but the swine isolates in this study were poorly recognized by this antiserum. This may be due to multiple amino acid substitutions located at Ca (K142R), Ca1 (S203T), and Sb (S185T) antigenic sites (Koel et al., 2015).

Three other 2019 isolates from the Pskov region were of H1 subtype belonging to the avian-like swine influenza lineage (H1avN2). To our knowledge, this is the first time such viruses have been detected in Russia.

The NA of the swine isolates from 2017 to 2019 belonged to the N2 subtype and was related to other N2 neuramidases identified in recent swine influenza A viruses in Europe (with an A/swine/Gent/1/1984 ancestor).

**FIGURE 5:**
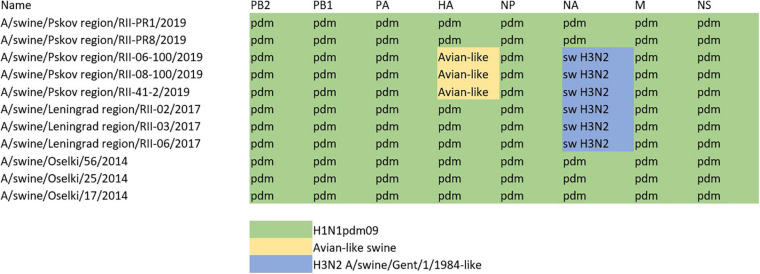
Genomic constellation of internal genes of swine influenza A(H1) viruses described within the study.

The constellation of internal genes of all sequenced H1 swine influenza viruses is presented in Figure 5. All swine influenza viruses identified within the study period had internal genes from pandemic A(H1N1)pdm09 human influenza viruses; this indicates that this subtype had been widely circulating in the swine population in Russia. This was further confirmed by serology studies of swine influenza sera collected throughout all years of the study.

The percent homology, between different swine HA of H1 subtype is presented in Supplementary Table 2. Supplementary Figure 1 shows HA phylogenetic trees for the H1 swine influenza strains. Supplementary Figures 2, 3 show phylogenetic trees for internal segments of swine influenza viruses described in the study. The genetic data was uploaded to the PubMed sequence read archive (SRA, Accession number PRJNA707597).

### Genomic Analysis of the Presence of Possible Virulence Markers or Substitutions Known to Confer Drug Resistance

Analysis of the possible presence of genetic markers of enhanced virulence did not reveal any mutations of special concern. All swine influenza H1N1pdm09, H1pdm09N2, and H1avN2 viruses identified in this study had internal genes of pandemic 2009 influenza strains with characteristic genomic signatures: truncated to 11 amino acid PB1-f2; glutamic acid at amino acid 627 of PB2; and S31N and R77Q in M2 protein, indicative of adamantane resistance. NA sequences were analyzed for marker mutations known to confer reduced or highly reduced sensitivity to the NA inhibitors, oseltamivir, and zanamavivir. PA sequences were analyzed for the presence of mutations known to confer baloxavir marboxil resistance. All viruses were sensitive to the NA inhibitors and baloxavir marboxil.

Genomic analysis of influenza A(H3N2) swine influenza isolated within this study revealed that they were human-like H3N2 viruses with all gene segments closely related to the human vaccine strain A/Perth/16/2009 and did not bear any markers of increased virulence. These strains also featured S31N in the M2 protein indicative of adamantane resistance (as human influenza H3N2 strains) and were sensitive to NA inhibitors and baloxavir marboxil.

### Serological Evidence of Influenza A Circulation in Swine

Over the study period, 1534 serum samples from swine were analyzed. Those serum samples were received from production sites where clinical signs of infection in animals were visible and from sites participating in monitoring without observable signs of ILI infection in animals. Serum samples positive for influenza A viruses of human and swine origin were detected in both types of production sites, indicating wide circulation of influenza strains in pigs.

**FIGURE 6:**
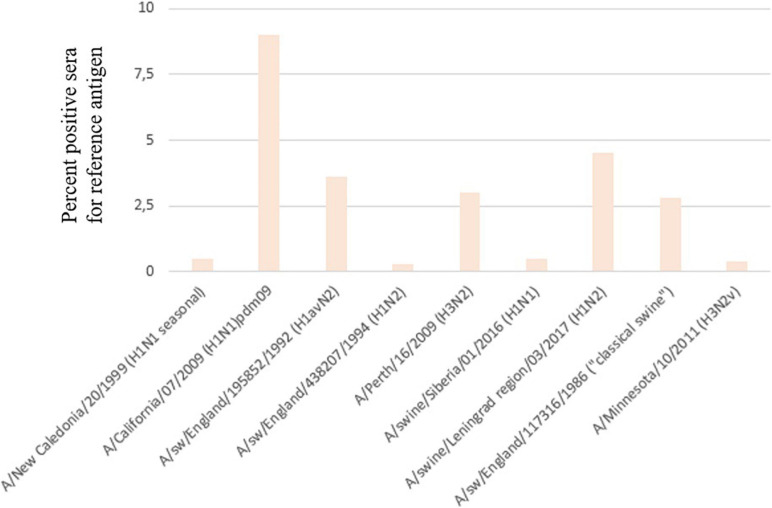
Percentage of serum samples that tested positive for reference antigens.

Serum samples were assessed by HI assay against a panel of human and swine antisera raised against reference viruses. The overall positive rate for each main antigen tested is presented in Figure 6. The majority of positive sera samples were detected for the A/California/07/2009, A/swine/England/195852/1992 (H1avN2), or A/swine/Leningrad region/03/2017 (H1pdm09N2) strains. This indicates that pandemic influenza A(H1N1)pdm09 strains and avian-like swine H1 strains widely circulate in pig population in Russia. A noticeable proportion of sera tested positive for the human A(H3N2) strain, although viruses of this subtype were isolated only in one season in 2014.

**FIGURE 7:**
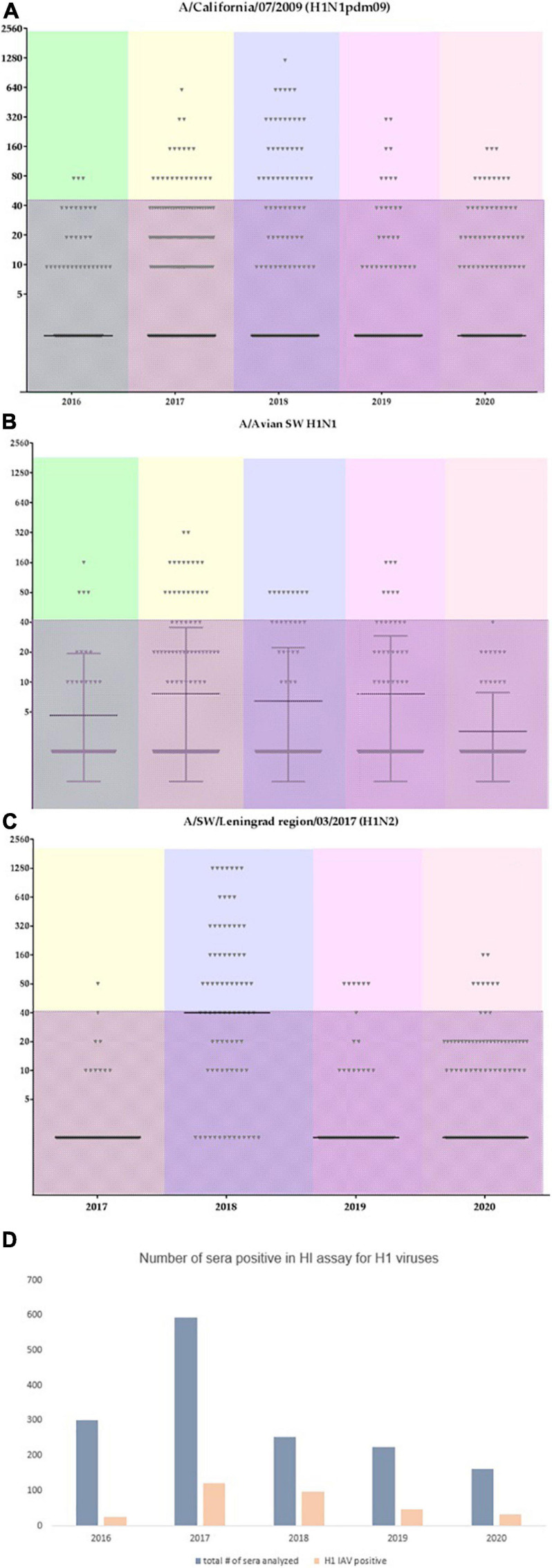
Individual serum titers from swine tested for the most abundant influenza A viruses. Color coding reflects different years. The shaded area marks titers with values of 40 or less. **(A)** individual titers to A/California/07/2009; **(B)** individual titers to A/swine/England/195852/1992 (H1avN2); **(C)** individual titers to A/swine/Leningrad region/03/2017 (H1N2); **(D)** overall number of sera tested within each year and the overall number of sera which tested positive for the influenza A(H1) subtype.

Figure 7 shows the distribution of HI assay titers of individual swine serum samples with the A/California/07/2009, A/swine/England/195852/1992 (H1avN2), and A/swine/Leningrad region/03/2017 (H1N2) strains in different time points (years of the study). Individual serum titers varied between animals. The shaded area marks low titer sera (40 or less) which cannot be considered as a sign of acute infection. Interestingly, the number of sera that tested positive for the influenza A/swine/England/195852/1992 (H1avN2) strain was lower last season in 2020 in comparison with previous seasons. However, the number of serum samples having titers against A(H1N1)pdm09 strains did not change. This could possibly indicate that the latter have more potential for sustained transmission and circulation in swine herds in the country.

## Discussion

Here, we report on genetic and antigenic characterization of swine influenza A viruses that were identified and isolated from swine herds in the European region of Russia. Our findings suggest that there are extensive signs of active influenza virus circulation in the country’s pig population; this matches previous reports regarding other European countries and Asia.

Serological monitoring of swine sera, from animals both with and without clinical signs of infection, has shown that both groups of animals have antibodies against influenza A viruses, predominantly against A(H1N1)pdm09. These viruses have been introduced into the swine population multiple times since the emergence of the 2009 pandemic. Antibodies to another influenza A subtype (H3N2) were found less frequently. The reasons for this are not entirely clear, as humans seem to be a major source of infection for animals at production sites. Influenza A(H3N2) viruses have been extensively circulating during Russia’s last 9 seasons. However, only a small fraction of swine samples features antibodies against human or swine viruses of this subtype.

Antigenic characterization of isolated strains showed that viruses isolated in 2014 were antigenically similar to the human vaccine strain A/California/07/2009; they grouped with viruses that were isolated at the beginning of A(H1N1)pdm09 human circulation in the country. However, viruses of this subtype isolated in later seasons were more antigenically distant and featured multiple substitutions in antigenic sites. The S183P amino acid change located near the receptor-binding site was a key marker of change for many human influenza A(H1N1)pdm09 viruses that was noticed early in the development of the 2009–2010 pandemic before spreading globally (WHO Influenza Centre London, 2011). D168N amino acid substitution, located at the Ca antigenic site, has also been observed in human and swine influenza A viruses multiple times (Tapia et al., 2020).

The antigenic diversity of influenza A(H1N1)pdm09 viruses has enhanced over time due to accumulation of multiple amino acid changes in the Ca, Sb, and Ca1 antigenic sites (Koel et al., 2015). All A(H1N1)pdm09 viruses isolated within the study grouped with the A(H1N1)pdm09 human and swine influenza viruses on phylogenetic trees, but showed pronounced antigenic differences in HI assay. This finding stresses the necessity and importance of carrying out antigenic analyses, in addition to genetic and phylogenetic analyses, to fully characterize circulating IAV strains.

Spread of A(H1N1)pdm09 viruses from humans back to the pig population caused the emergence of multiple reassortant genotypes, many of which are now well-established in different parts of the world (Lewis et al., 2016; Henritzi et al., 2020; Anderson et al., 2021). The European ESNIP 3 project and a follow-up study (Watson et al., 2015; Henritzi et al., 2020) have shown that the prevalence of IAV-infected swine farms has been growing substantially in recent years, and our study is in line with this observation. Two reassortant lineages described earlier in Europe, H1pdmN2, and H1avN2, were also identified in the European region of Russia. This gives additional evidence that the number of swine influenza A reassortant viruses bearing internal genes from H1pdm09 is growing. The H1pdmN2 strains described in this study were antigenically distinct from human A(H1N1)pdm09 strains, indicating that immunity via influenza vaccination may not be protective against these viruses.

We were also able to isolate and characterize swine influenza viruses that had avian-like H1 hemagglutinin. This is the first time, to our knowledge, that such viruses have been identified in Russia, although such strains have been described in other countries in numerous studies (Watson et al., 2015; Feng et al., 2020; Henritzi et al., 2020; Sun et al., 2020). Eurasian avian-like (EA) viruses widely circulate in Europe (Henritzi et al., 2020; Zell et al., 2020) and China (Feng et al., 2020) and recently multiple diverse genotypes of Eurasian avian-like H1N1 viruses were identified in China with the G4 EA genotype being of special concern (Sun et al., 2020). Our studies indicate that the reassortant swine influenza H1avN2 strains isolated in this study do not belong to this genotype and bear all internal genes from pandemic H1N1pdm09 virus.

The genetic backbone of influenza A strains of A(H1) subtype identified here supports the idea that all identified reassortment variants were the result of reassortment events that took place within swine herds at production sites, or possibly during periods of transportation of animals between sites. The fact that two different swine influenza A viruses (H1pdm09 and H1avN2) were isolated from the same production site gives additional evidence that reassortment events took place within swine herds at production sites.

**FIGURE 8:**
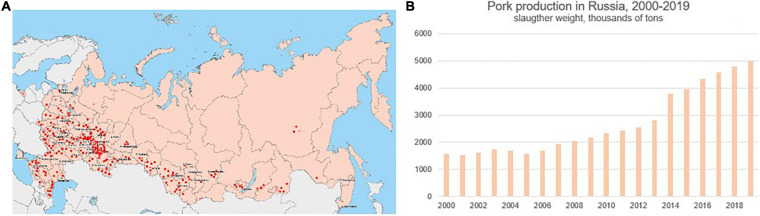
Distribution of swine (pork) production sites in Russia **(A)**, red dots, and pork production volume in Russia from 2000 to 2019 **(B)**, according to open data from the National Swine Producers Union (http://www.nssrf.ru/).

It should be noted that there are two major factors that favor influenza spread in swine herds. Firstly, pork production has grown rapidly over the last 20 years (Figure 8), and pork producing sites are not distributed equally across the country. They are densely located in the European geographic region. Secondly, pigs are not vaccinated against influenza in Russia, making them highly susceptible to the infection at all stages of the production cycle. Given that the swine influenza strains identified by this study were antigenically distinct from those circulating in humans, and carry antigenically-distinct H1pdm09, avian-like H1, and human-unrelated N2 segments, it is important to conduct regular swine surveillance for influenza in the country.

The diversity of swine influenza strains in Russia remains largely unexplored (Chauhan and Gordon, 2020) due to an absence of well-establish surveillance. These conditions make this study of influenza viruses circulating in the population of pigs in the European region of the country highly relevant. Swine influenza viruses are infecting humans (Bowman et al., 2017; Meijer et al., 2018; Deng et al., 2020), and given their ability to undergo frequent reassortment, it is impossible to predict in advance the properties of newly emerging strains.

This study has multiple limitations. First, all samples were collected only in the European region of the country and thus cannot reveal geographical patterns of IAV circulation nationally. Secondly, the sampling of pig production sites was not equal in different years of this study, and we were able to isolate viruses only in the three seasons of 2014, 2017, and 2019. The number of viruses isolated was also limited. Sequencing was carried out only for isolated strains. However, many PCR positive samples were not sequenced. Lastly, serological studies of influenza A antibodies in the swine population indicate wide circulation of these pathogens in the country’s swine herds and underscore the need for continuous IAV surveillance in the country.

In this regard, continuous further studies must be carried out: to determine the antigenic, genetic and biological properties of these viruses; to provide better understanding of their major characteristics that might affect the human population; and to enable timely assessment of their virulence and pandemic potential. Our results describe swine influenza viruses identified in Russia, while contributing to global knowledge regarding influenza A in both humans and animals.

## Data Availability Statement

The datasets presented in this study can be found in online repositories. The names of the repository/repositories and accession number(s) can be found in the article/Supplementary Material.

## Ethics Statement

The animal study was reviewed and approved by the Local Bioethics Committee, Smorodintsev Research Institute of Influenza.

## Author Contributions

DD and AK designed the study, took part in all experimental work, analyzed the results, and wrote the draft of the manuscript. AF, MB, AI, and KK performed PCR and sequencing, and analyzed results. PP and AVs performed virus isolation, antigenic characterisation, and performed antigenic cartography. AZ and NK performed serological analysis of swine sera and analyzed results. AVi analyzed results and prepared the draft of the manuscript. All authors have read and approved the final text of the manuscript.

## Conflict of Interest

The authors declare that the research was conducted in the absence of any commercial or financial relationships that could be construed as a potential conflict of interest.

## Footnotes

1 http://acmacs-web.antigenic-cartography.org

## Supplementary Material

Supplementary Figure 1 Maximum likelihood HA phylogenetic trees for the H1 swine influenza strains isolated in the study.

Supplementary Figure 2 Maximum likelihood phylogenetic trees for internal segments of H1 swine influenza viruses described in the study.

Supplementary Figure 3 Maximum likelihood phylogenetic trees for internal segments of H3 swine influenza viruses described in the study.

Supplementary Table 1 GISAID accession numbers of viruses described in the study.

Supplementary Table 2 HA homology between different swine IAV strains.
