# Supplementary figures and images for: Antigenic and Genetic Characterization of Swine Influenza Viruses Identified in the European Region of Russia, 2014–2020
Source: Front Microbiol. 2021 Apr 15;12:662028. doi: 10.3389/fmicb.2021.662028 (PMC8081852; doi:10.3389/fmicb.2021.662028)

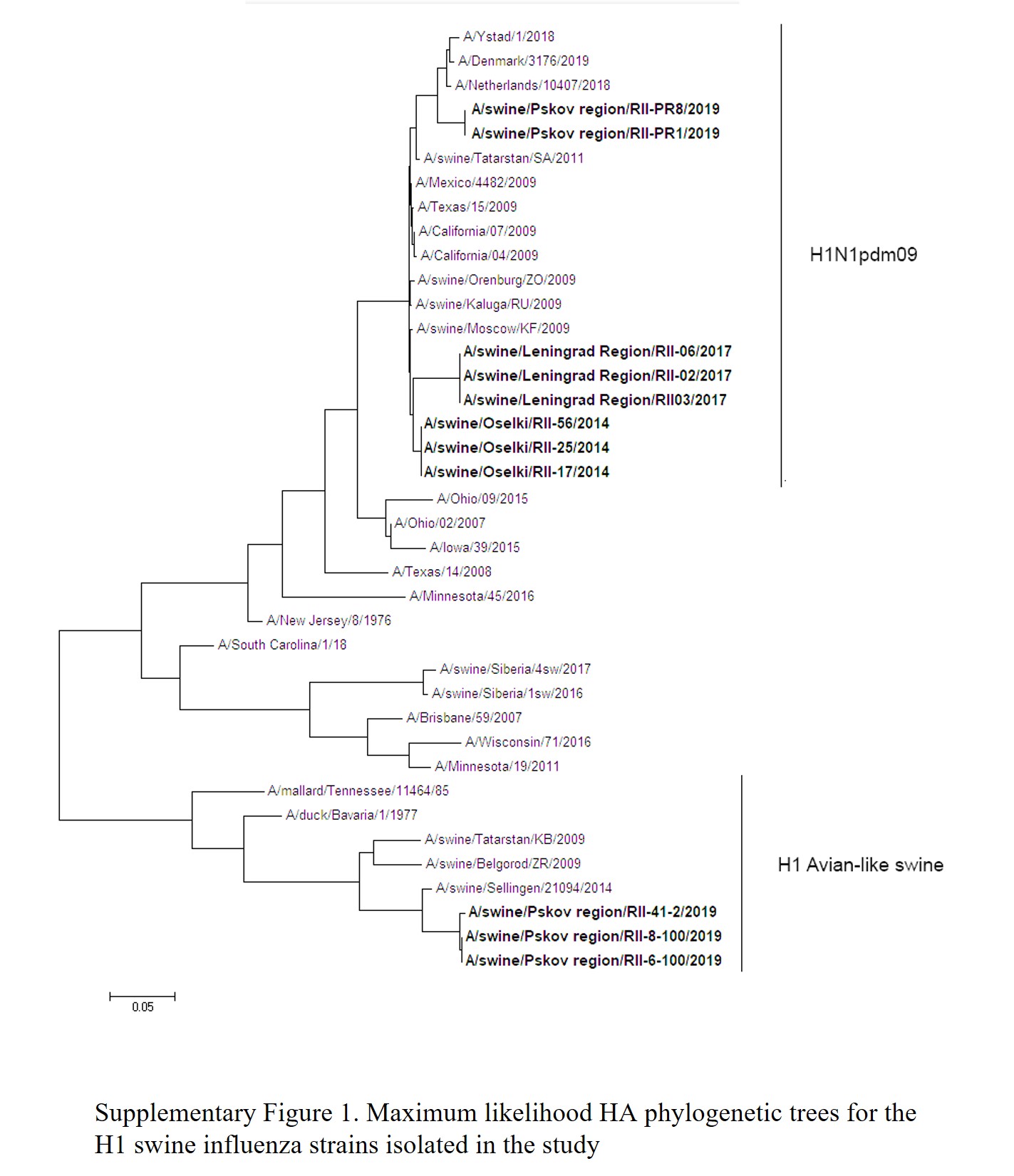

Supplement: Supplementary Figure 1 — Maximum likelihood HA phylogenetic trees for the H1 swine influenza strains isolated in the study. [file Image_1.JPEG]

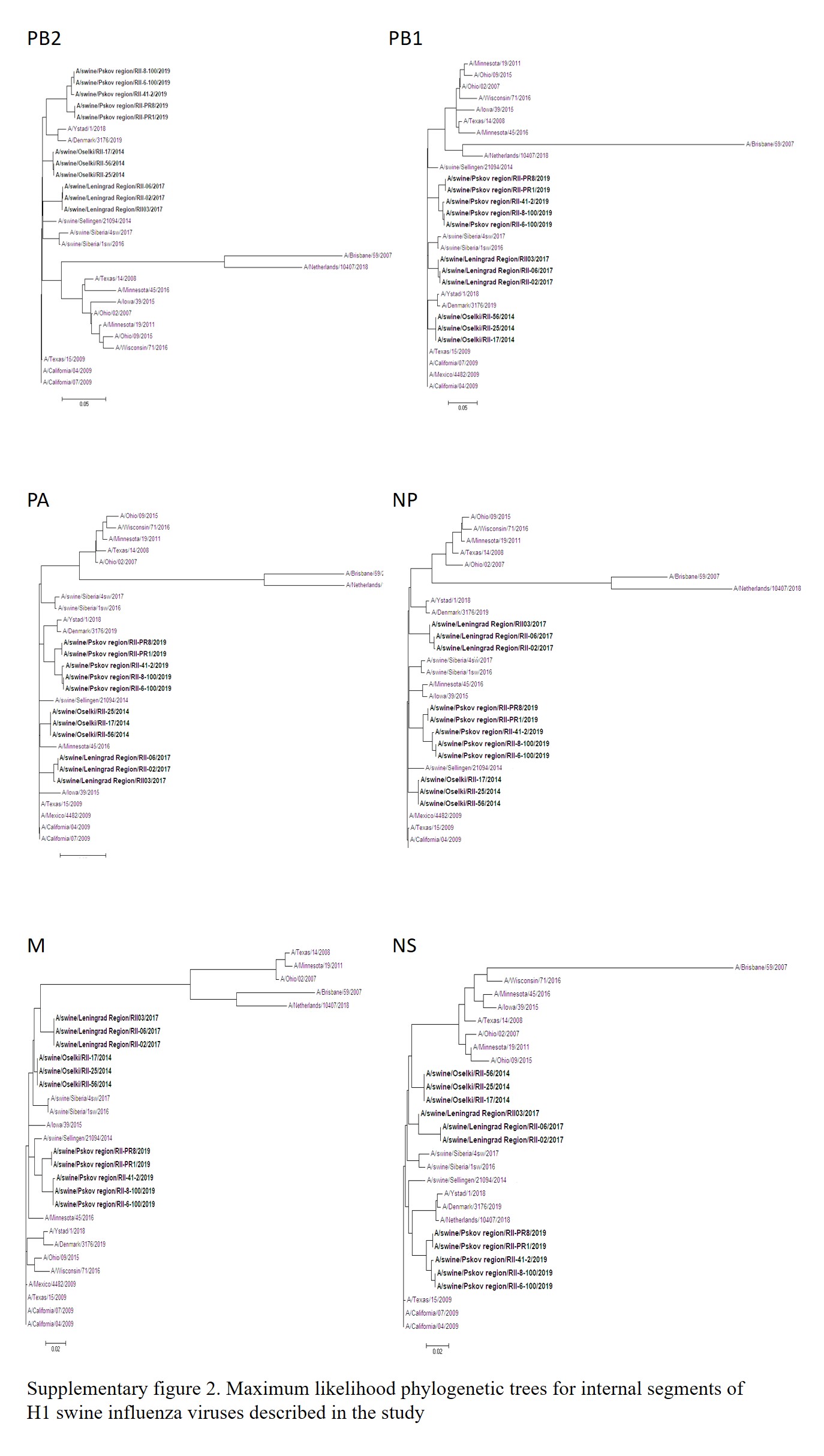

Supplement: Supplementary Figure 2 — Maximum likelihood phylogenetic trees for internal segments of H1 swine influenza viruses described in the study. [file Image_2.JPEG]

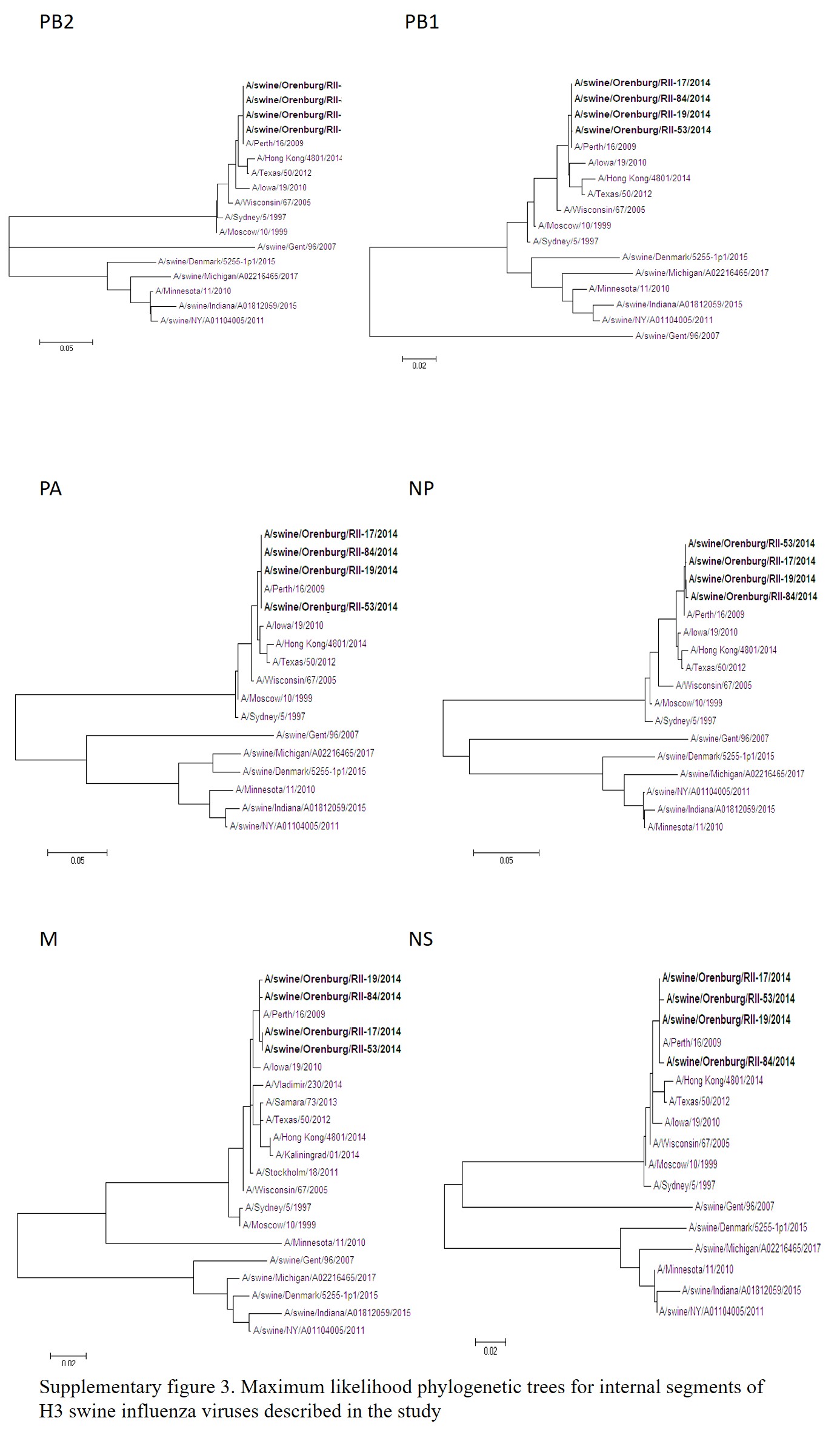

Supplement: Supplementary Figure 3 — Maximum likelihood phylogenetic trees for internal segments of H3 swine influenza viruses described in the study. [file Image_3.JPEG]
