# Supplementary material for: Antigenic and Genetic Characterization of Swine Influenza Viruses Identified in the European Region of Russia, 2014–2020
Source: Front Microbiol. 2021 Apr 15;12:662028. doi: 10.3389/fmicb.2021.662028 (PMC8081852; doi:10.3389/fmicb.2021.662028)
Supplement: Supplementary Table 1 — GISAID accession numbers of viruses described in the study. [file Table_1.DOCX]

Supplementary table 1. GISAID accession numbers of the viruses described in the study

| Strain name | GISAID accession number |
| --- | --- |
| A/sw/Oselki/RII-17/2014 | EPI_ISL_906137 |
| A/sw/Oselki/RII-25/2014 | EPI_ISL_906138 |
| A/sw/Oselki/RII-56/2014 | EPI_ISL_906139 |
| A/sw/Orenburg/RII-17/2014 | EPI_ISL_906142 |
| A/sw/Orenburg/RII-19/2014 | EPI_ISL_906143 |
| A/sw/Orenburg/RII-53/2014 | EPI_ISL_906141 |
| A/sw/Orenburg/RII-84/2014 | EPI_ISL_906140 |
| A/sw/Leningrad region/RII-02/2017 | EPI_ISL_1095455 |
| A/sw/Leningrad region/RII-03/2017 | EPI_ISL_314323 |
| A/sw/Leningrad region/RII-06/2017 | EPI_ISL_1096143 |
| A/sw/Pskov region/RII-06-100/2019 | EPI_ISL_906134 |
| A/sw/Pskov region/RII-08-100/2019 | EPI_ISL_906135 |
| A/sw/Pskov region/RII-41-2/2019 | EPI_ISL_906136 |
| A/sw/Pskov region/RII-PR1/2019 | EPI_ISL_906132 |
| A/sw/Pskov region/RII-PR8/2019 | EPI_ISL_906133 |
